# Percutaneous extracorporeal ventricular assist device as a successful bridge strategy to durable left ventricular assist device in refractory cardiogenic shock: a case report

**DOI:** 10.3389/fcvm.2026.1720250

**Published:** 2026-05-12

**Authors:** Dan Li, Jianfeng Chen, Jinping Liu, Jinping Zhao

**Affiliations:** 1 Department of Cardiovascular Surgery, Zhongnan Hospital of Wuhan University, Wuhan, Hubei, China; 2 Department of Cardiovascular Surgery, Hubei Provincial Engineering Research Center of Minimally Invasive Cardiovascular Surgery, Wuhan, Hubei, China; 3 Department of Cardiovascular Surgery, Wuhan Clinical Research Center for Minimally Invasive Treatment of Structural Heart Disease, Wuhan, Hubei, China

**Keywords:** cardiogenic shock, left ventricular assist device, mechanical circulatory support, percutaneous, pulmonary edema

## Abstract

**Introduction:**

Cardiogenic shock (CS) is a life-threatening condition with high mortality. Temporary mechanical circulatory support (tMCS) is important for stabilizing patients with refractory CS, acting as a bridge to recovery, decision-making for durable left ventricular assist device (LVAD) implantation or transplantation. In China, commonly used devices like intra-aortic balloon pumps (IABP) and extracorporeal membrane oxygenation (ECMO) have limitations such as inadequate ventricular unloading and high complication rates, and advanced options like Impella, TandemHeart and CentriMag are not widely available. This report presents a successful bridge to durable LVAD therapy case using a minimally invasive extracorporeal VAD in a high-risk CS patient.

**Case summary:**

A 39-year-old male with refractory cardiogenic shock due to ischemic cardiomyopathy is reported. He was not a candidate for immediate durable LVAD implantation or transplantation because of unstable hemodynamics, severe pulmonary edema, and active infection. Considering his condition, a percutaneous extracorporeal VAD was inserted through the right axillary artery and jugular vein, creating a bypass from the left atrium → across the atrial septum → right atrium → superior vena cava → right jugular vein → centrifugal pump → axillary artery. The operation was carried out without any complications, and the patient quickly improved. After 215 h and 43 min of support, he successfully received durable LVAD implantation.

**Discussion:**

This case demonstrates the clinical value of a minimally invasive extracorporeal VAD as a bridge-to-bridge therapy for critical cardiogenic shock. The percutaneous approach effectively unloads the left ventricle while minimizing complications. It promotes early patient mobilization, simplifies postoperative care, and lowers treatment costs. This method is technically simple and controllable in most hospital environments, offering practical guidance for managing high-risk shock patients, especially in places where advanced surgical options are restricted.

## 1 Introduction

Cardiogenic shock (CS) is a complex clinical syndrome resulting from a significant reduction in cardiac output due to left, right, or biventricular dysfunction, which leads to pulmonary edema and severe hypoperfusion of organs and tissues (1–3). When patients are too unstable to endure durable left ventricular assist device (LVAD) implantation or heart transplantation surgery and traditional therapies are ineffective, bridging strategies become essential, particularly for patients with refractory heart failure (4). Early application of temporary mechanical circulatory support (tMCS) can significantly improve outcomes by augmenting cardiac output, improving coronary perfusion, reducing ventricular afterload, and decreasing left ventricular filling pressures. These devices play an important role as a bridge to recovery, decision-making, durable ventricular assist device implantation, or heart transplantation (5–7).

In China, the most commonly used temporary support devices include extracorporeal membrane oxygenation (ECMO) and intra-aortic balloon pumps (IABP) (8). However, these devices provide only limited relief of left ventricular pressure, and their prolonged use raises the risk of complications such as hemolysis, limb ischemia, bleeding, and thrombosis. Alternative options like Impella, TandemHeart and CentriMag are not commercially available in China, limiting therapeutic choices.

Here, we report a case of refractory cardiogenic shock that was successfully managed with a percutaneous extracorporeal ventricular assist device, implanted via the right jugular vein and axillary artery. This technique avoided the need for sternotomy, promoted early rehabilitation, and successfully bridged the patient to a durable LVAD. The approach is straightforward, feasible for widespread hospital use, and offers meaningful clinical benefits.

## 2 Case presentation

The patient was a 39-year-old male (height: 165 cm, weight: 58 kg) with end-stage ischemic cardiomyopathy, presenting to our medical center with acute decompensated heart failure that was refractory to inotropic support. His underlying medical history included dyslipidemia, but he did not take lipid-lowering drugs regularly. He had no history of hypertension, diabetes, smoking, or alcohol consumption. Two months ago, he experienced an acute myocardial infarction (AMI) with cardiogenic shock and was admitted to another hospital. Treatments included percutaneous coronary intervention (PCI), venoarterial ECMO (VA-ECMO, weaned after 10 days), and IABP (weaned after 20 days). Despite receiving maximal medical therapy at multiple centers, he still had recurrent chest pain, dyspnea, and severely limited exercise tolerance. Following his transfer to our institution, he was diagnosed with refractory heart failure (NYHA class IV), a large apical left ventricular aneurysm, persistent pulmonary edema and pneumonia.

The transthoracic echocardiogram revealed severe left ventricular dysfunction (LVEF 25%). Additionally, a large apical aneurysm and moderate mitral regurgitation were observed. The coronary angiography showed patent stents without significant new lesions, eliminating a target for further surgical revascularization. He had received long-term standardized medical therapy, including positive inotropic agents, vasopressors, diuretics, ß-blockers, ACE inhibitors, statins, and dual antiplatelet therapy. However, he remained hemodynamically unstable with impaired mobility, presenting with low blood pressure and severe pulmonary edema. Despite recieving high-dose norepinephrine (0.08*μ*g/kg/min) and dobutamine(10.3μg/kg/min), his lowest recorded blood pressure was 78/45mmHg, and his heart rate was 118bpm. Under high-flow oxygen therapy, his peripheral oxygen saturation (SpO_2_) was 90% with a minimum partial pressure of oxygen (PO_2_) of 58mmHg. The lactate level was 2.0 mmol/L. Additionally, he suffered from a continuous fever that peaked at 38.5 °C, along with cold extremities. Invasive hemodynamic monitoring via Swan-Ganz catheter revealed a pulmonary capillary wedge pressure (PCWP) of 36 mmHg. He was classified as SCAI stage D and INTERMACS profile 1–2. The efficacy of the anti-infective treatment was limited due to severe pulmonary edema. Given these reasons, the multidisciplinary team (MDT) concluded that immediate durable LVAD implantation or transplantation carried a prohibitively high risk due to his unstable hemodynamics, severe pulmonary edema, and active infection. Thus, a bridge-to-decision strategy was adopted, aiming to alleviate pulmonary congestion and stabilize hemodynamics with extracorporeal VAD.

The extracorporeal VAD system was composed of a centrifugal pump (Rotaflow, Maquet) and the associated tubing. Our team contributed the patient's progessively worsening hypoxemia to severe pulmonary edema secondary to left heart failure. Since the circuit drew blood from the left atrium, which contains oxygenated blood, our team considered that an oxygenator was unnecessary. It was the key distinction from ECMO system, and it also reflected the objective of providing ventricular unloading rather than respiratory support. The extracorporeal VAD system was connected through cannulation of the right axillary artery and right internal jugular vein for postoperative recovery, early mobilization, and hemodynamic stability.

**Figure 1:**
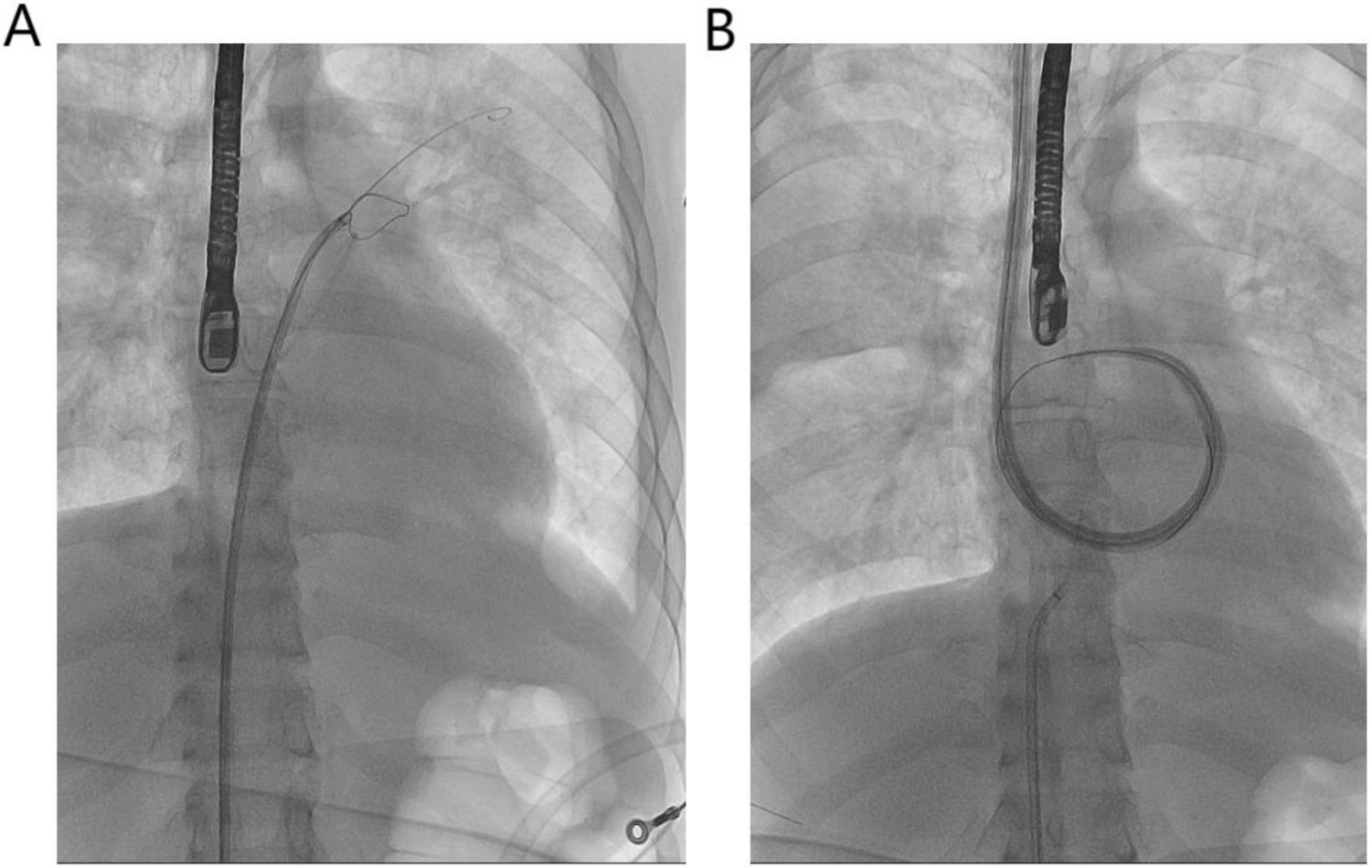
Jugular vein catheterization procedure. **(A)** DSA image showing a snare introduced via the right femoral vein, capturing a guidewire advanced from the right jugular vein and directing it into the left atrium. **(B)** DSA image confirming the final position of the drainage cannula in the left atrium via the established trans-septal pathway.

An 8-mm vascular graft (Maquet) was anastomosed to the axillary artery and connected to a Medtronic DLP 77418 cannula for arterial inflow. A long, single stage, multi-sidehole drainage cannula (Medtronic, Bio-Medicus Life Support™ Flex XL, LS-96555-019) was inserted into the right jugular vein for outflow. The cannula with side holes limited to the distal 3 cm. The objective was to position the dispal cannula containing the side holes entirely in the left atrium to achieve the directly left ventricular unloading. We initially tried to insert the catheter through the jugular vein, but found it too difficult, so we switched to the right femoral vein approach. Under digital subtraction angiography (DSA) and ultrasound guidance, a guidewire was inserted through the right femoral vein, and a snare was placed in the left atrium along the wire. The guidewire was advanced through the jugular vein, captured by the snare, and guided across the atrial septum into the left atrium. After establishing this pathway, the drainage cannula was advanced over the wire and positioned in the left atrium (Figure 1). Finally, this established an extracorporeal circuit pathway from the left atrium→ across the atrial septum→right atrium→superior vena cava→right jugular vein→ centrifugal pump (Maquet, Rotaflow) → returning to the axillary artery.

**Figure 2:**
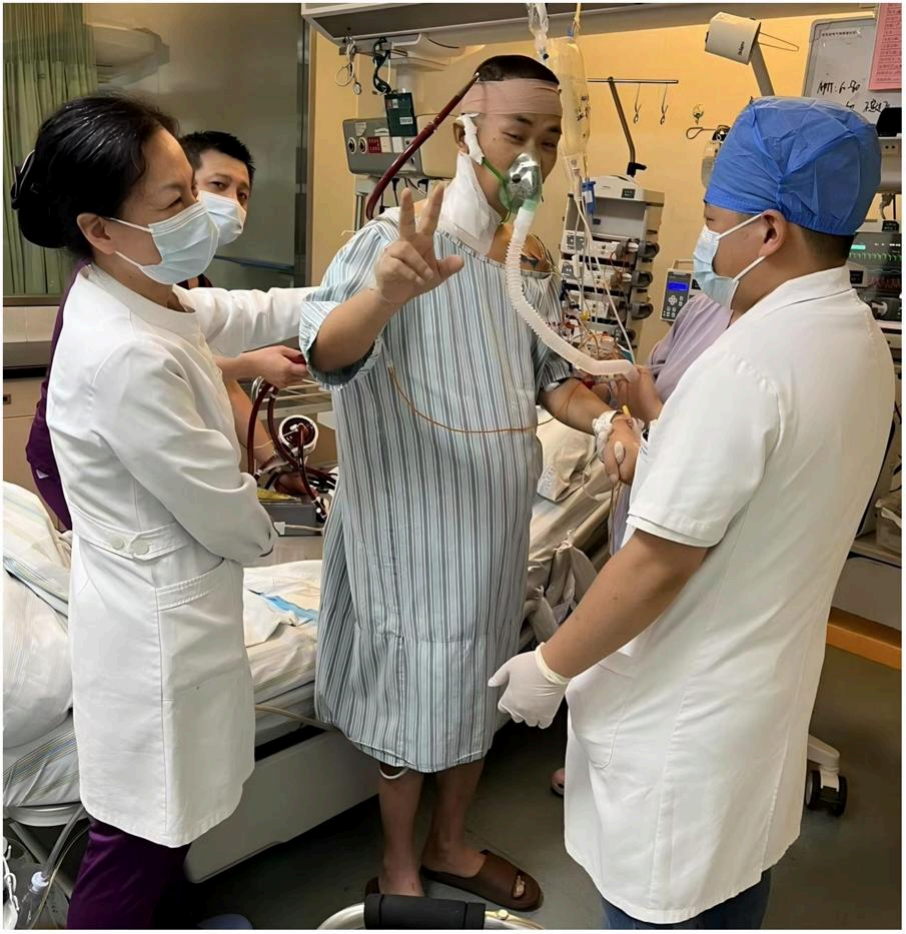
The patient performing bedside exercises while on extracorporeal VAD support, demonstrating the potential for early mobilization with this percutaneous approach.

**Figure 3:**
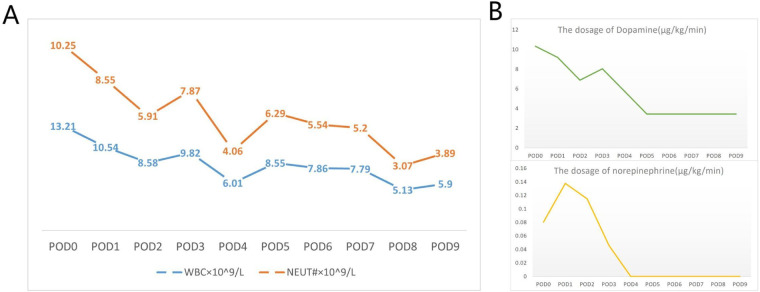
Trends in the patient's clinical status during extracorporeal VAD support. **(A)** The trend of infection indicators: white blood cell count (WBC, red line) and absolute neutrophil count (NEUT, orange line) over time, both showing a trend towards normalization. **(B)** The dosage of vasopressors: Dopamine (green line) and Norepinephrine (yellow line) infusion rates over time, showing a substantial decrease.

**Figure 4:**
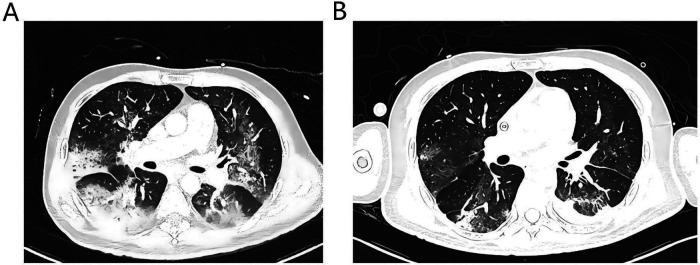
Comparative chest CT scans: **(A)** preoperative CT scan showing extensive bilateral pulmonary edema and consolidation. **(B)** CT scan on the eighth day of extracorporeal VAD support showing significant resolution of pulmonary edema and consolidation.

The operation was completed successfully without any complications. Argatroban was used for anticoagulation, aiming for an APTT of 60–80 s. The initial pump flow was set at 2.2 L/min but was later decreased to 1.8–2.0 L/min due to indications of right upper limb hyperperfusion, such as heightened warmth and slight swelling. These symptoms quickly subsided after the flow was reduced, and the need for vasopressors remained consistent during this adjustment.To reduce the risk of migration, the cannula was firmly fixed at the cutaneous insertion point using sutures. The external tubing was further stabilized with an elastic bandage wrapped around the head. The cannula position was confirmed by chest radiography and remained stable during the period of mechanical support. The patient recovered rapidly after the procedure. He was extubated on the first postoperative day, began oral intake, and started physical therapy at his bedside (Figure 2). Under low flow oxygen therapy, his SpO_2_ was sustained above 95%, with the minimum recorded PaO_2_ exceeding 100 mmHg. The patient's condition was progressively getting better, as indicated by the normalization of infection markers and a substantial decrease in the vasopressor dosage (Figure 3). Serial chest radiographs and a CT scan on the eighth day showed significant improvement in his pulmonary edema and pneumonia (Figure 4). The extracorporeal VAD system provided 215 h and 43 min of effective circulatory support as a bridge-to-bridge strategy. After multidisciplinary evaluation, the patient successfully underwent durable LVAD implantation.

## 3 Discussion

Cardiogenic shock (CS) is a condition marked by low cardiac output, leading to hypoperfusion. It may result from acute cardiac events—whether ischemic or non-ischemic—or worsen from underlying chronic heart disease (1, 3). Despite advances in management strategies, cardiogenic shock continues to carry poor prognosis, with contemporary studies reporting in-hospital mortality rates of 27%–51% (3, 9).

The application of temporary mechanical circulatory support devices has become a critical strategy in stabilizing hemodynamics and improving outcomes (10). It can provide hemodynamic assistance for periods ranging from hours to weeks. Selecting and implanting a suitable tMCS device in a timely manner is critical, as it can alleviate hemodynamic compromise, facilitate partial or complete cardiac unloading, and thereby improve clinical outcomes in critically ill patients. Temporary mechanical circulatory support plays a vital role as a bridge to three key goals: clinical decision-making, myocardial recovery, or destination therapy (transplantation or long-term mechanical support) (6, 11).

The most commonly used tMCS devices in China are IABP and ECMO. IABP is simple to operate and can provide hemodynamic benefits such as reduced myocardial oxygen consumption, increased coronary artery perfusion, decreased afterload, and a slight increase in cardiac output (0.8–1.0 L/min) (1, 12). However, its efficacy in left ventricular unloading is extremely limited and debate continues over the use of IABP in patients with CS. The European guidelines have downgraded IABP recommendations, and now it is only recommended in CS patients of Class IIIB (13). A basic restriction of ECMO is its incapacity to offer ventricular unloading, even though it supplies considerable circulatory and respiratory assistance. In patients undergoing femoral artery cannulation, retrograde aortic blood flow could increase left ventricular end-diastolic pressure. This diminishes coronary blood supply and raises myocardial oxygen demand, causing serious issues like pulmonary edema or thrombosis from left ventricular stasis. The severity of these complications shows a flow-dependent relationship, intensifying as the ECMO outputs increase (14). In cardiogenic shock patients, ECPella—a combined tMCS strategy that integrates ECMO for circulatory and respiratory support and Impella for direct left ventricular unloading, has shown promising clinical outcomes, particularly in demonstrating a significant reduction in 30-day mortality rates. However, the combination of two different tMCS devices unavoidably leads to an increased incidence of complications (15). Meanwhile, Impella is still in the early stages of adoption in China. Other options, such as TandemHeart and CentriMag, are not commercially available. It's also important to note that CentriMag and the surgical Extra-VAD system both require sternotomy for implantation (16). Therefore, the key challenge in the current Chinese clinical setting is to identify a simple, effective, and easily implantable tMCS device that can be widely adopted across most medical centers and offer sufficient ventricular unloading without needing invasive surgical operation or complex combinations.

In present clinical practice, the Society for Cardiovascular Angiography and Interventions (SCAI) shock classification and Interagency Registry for Mechanically Assisted Circulatory Support (INTERMACS) profile are the most commonly used among the various methods for characterizing the severity of cardiogenic shock (10, 17). They serve as reliable tools for risk stratification and mortality prediction. The patient exhibited progressive shock refractory to escalated medical therapy, meeting criteria for both SCAI stage D and INTERMACS profile 1–2. According to literature reports, INTERMACS profile 1 patients undergoing immediate durable LVAD implantation face a 30-day mortality of 20% and a 2-year mortality rate reaches as high as 50%. Nevertheless, these patients are not excluded from durable LVAD implantation. It is suggested that such patients focus on temporary mechanical circulatory support as a bridge therapy (18).

In this case, we successfully stabilized a critically ill patient (SCAI D, INTERMACS 1–2) by resolving life-threatening pulmonary edema, reducing accidental complications, and enabling early rehabilitation therapy. The extracorporeal VAD employed provided sufficient flow and effective left ventricular unloading, sharing the same physiological objective as dedicated percutaneous left ventricular assist devices such as the TandemHeart. However, our system offers several distinct practical advantages in China when compared with TandemHeart and Impella, especially in resource-limited environments. First, in terms of accessibility and technical simplicity, TandemHeart and Impella are not commercially available in China, which severely limits their immediate clinical utility. In contrast, our system consists solely of a centrifugal pump and tubings with a straightforward design, which enhances its wide accessibility and feasibility for rapid deployment in most hospital settings. Second, it is also important to note that limb ischemia, hemolysis, thrombosis, and bleeding are common complications associated with tMCS, including TandemHeart, and the extended duration of support correlates with a higher incidence of these complications (15, 19). In the patient, axillary artery cannulation via vascular graft effectively prevents distal limb ischemia.Unlike the Impella, which requires crossing the aortic valve and poses risks of valvular injury or hemolysis, our system potentially reducing these risks. During the procedure, advanced imaging guidance guarantees precise sheath placement and reduces the risk of implant malposition. Third, in terms of rehabilitation and invasiveness, our approach offers notable benefits. The percutaneous transvenous catheter approach enables early postoperative ambulation of the patients, accelerating functional recovery and rehabilitation while reducing the incidence of postoperative bed rest–associated complications (such as deep vein thrombosis and lung infections), and simplifying postoperative management. This approach offers an additional advantage compared to Tandemheart, as the conventional femoral veno-arterial cannulation strategy restricts patient ambulation and complicates early rehabilitation. Fourth, in terms of flexibility and cost-effectiveness, our system can also enable immediate conversion to full venoarterial ECMO support by adding an oxygenator into the existing circuit in the event of of right heart failure or severe hypoxia. Additionally, its simpler structure also leads to a lower cost compared to other devices, making it a more cost-effective choice with a diminished economic burden. This cost advantage is particularly critical in clinical settings where medical resources are limited.

## 4 Conclusion

In conclusion, this minimally invasive approach serves as a key bridging strategy, particularly when patients require urgent circulatory support but are not immediate candidates for durable LVAD or heart transplantation. The technique provides hemodynamic stability while reducing organ congestion, helping to preserve vital function and optimize conditions for eventual permanent device implantation. This technique allows for rapid deployment with minimal trauma, making it suitable for high-risk patients. The procedure is simple to perform and relatively easy to manage, feasible for most medical centers, and cost-effective, easing financial burdens while accelerating recovery. These practical advantages offer significant guidance for clinical practice, particularly in managing critically ill cardiogenic shock patients.

## Data Availability

The original contributions presented in the study are included in the article/Supplementary Material, further inquiries can be directed to the corresponding authors.
